# Case of a 96‐year‐old woman with tilt of the subjective vertical axis

**DOI:** 10.1002/acn3.70003

**Published:** 2025-02-11

**Authors:** Markus A. Hobert, Patrik Theodor Nerdal, Klaus Jahn, Johannes Hensler, Walter Maetzler

**Affiliations:** ^1^ Department of Neurology University Hospital Schleswig‐Holstein, and Christian‐Albrechts‐University of Kiel Kiel Germany; ^2^ Department of Neurology Schoen Clinic Bad Aibling Bad Aibling Germany; ^3^ Department of Geriatrics Schoen Clinic Bad Aibling Bad Aibling Germany; ^4^ German Center for Vertigo and Balance Disorders Ludwig‐Maximilians‐University of Munich Munich Germany; ^5^ Department of Neuroradiology University Hospital Schleswig‐Holstein, and Christian‐Albrechts‐University of Kiel Kiel Germany

interACTN Case #44: Available: https://interactn.org/2025/01/30/case‐44‐the‐case‐of‐a‐96‐year‐old‐woman‐with‐tilt‐of‐subjective‐vertical‐axis/

## Summary of Case (HPI, Relevant Exam Findings, and Relevant Data)

A 96‐year‐old woman presents to the University Hospital Emergency Department by ambulance with hypertension and a tendency to fall to the left. The latter symptom had been present for 11 days and had worsened in the last few days. On clinical examination, the patient had a blood pressure of 230/110 mmHg, a tendency to fall to the left, and an inability to walk independently. Testing the subjective visual vertical (SVV) perception with the bucket test, the vertical visual axis was tilted about 20 degrees to the left. Brain MRI showed an ischemic infarct in the left dorsal spinocerebellar tract responsible for the SVV tilt.^1^, ^2^ The patient was transferred to the neuro‐geriatric unit for 2 weeks. Early rehabilitation geriatric complex treatment focused on training of vertical axis perception and gait. She could be discharged home without any need for additional support.

A tilt of the SVV can be caused by central lesions in different locations. The main structures in the network for verticality perception are graviceptive pathways running from the inner ear via vestibular nuclei, midline midbrain, the dorsolateral thalamus to the parieto‐insular vestibular cortex (PIVC). Besides the vestibular system, a modulating network of different cerebral structures that integrates visual, spinal, and cerebellar information contributes to verticality perception. Depending on the lesion site, the tilt of the SVV can be ipsilateral (medullary brainstem lesions) or contralateral (midbrain lesions). Lesions of the vestibular thalamus, cerebellum, vestibulo‐cerebellar tracts, and cortical areas can cause ipsilateral or contralateral tilt.^1^

## Diagnosis

**Figure 1:**
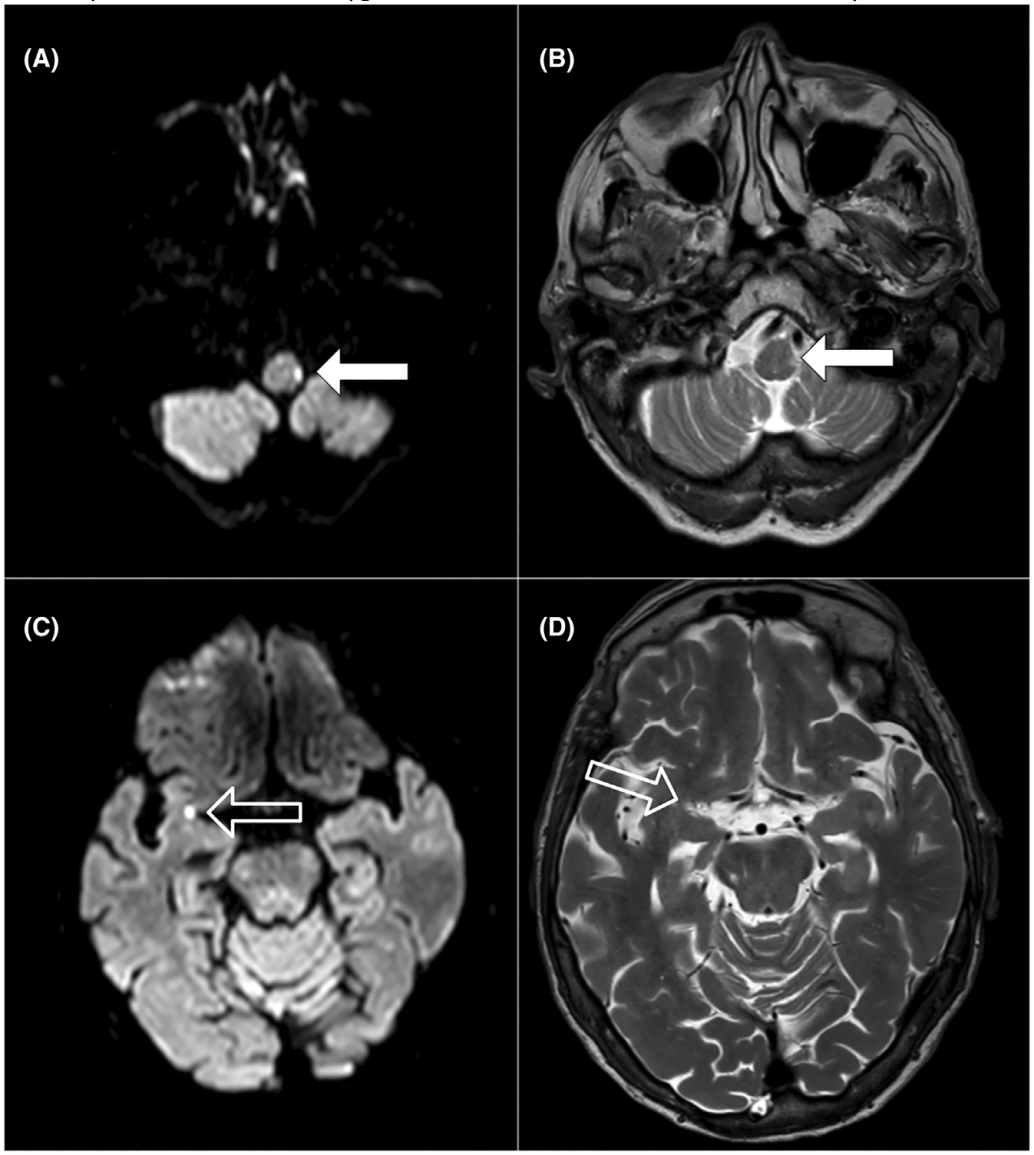
MRI (3T, Ingenia CX, Philips) acquired 14 days after symptom onset demonstrates acute ischemic lesions with diffusion‐weighted imaging (DWI) restriction and T2‐weighted (T2W) hyperintensity in the left medullary region (white arrows A and B) and in the right subinsular region (translucent arrows C and D).

Ischemic infarcts in the left dorsal spinocerebellar tract and subinsular right (see Fig. 1) close to the parieto‐insular vestibular cortex (PIVC). The medullary lesion was likely causing the tilt in verticality perception.^2^

## Take‐Home Points

**Figure 2:**
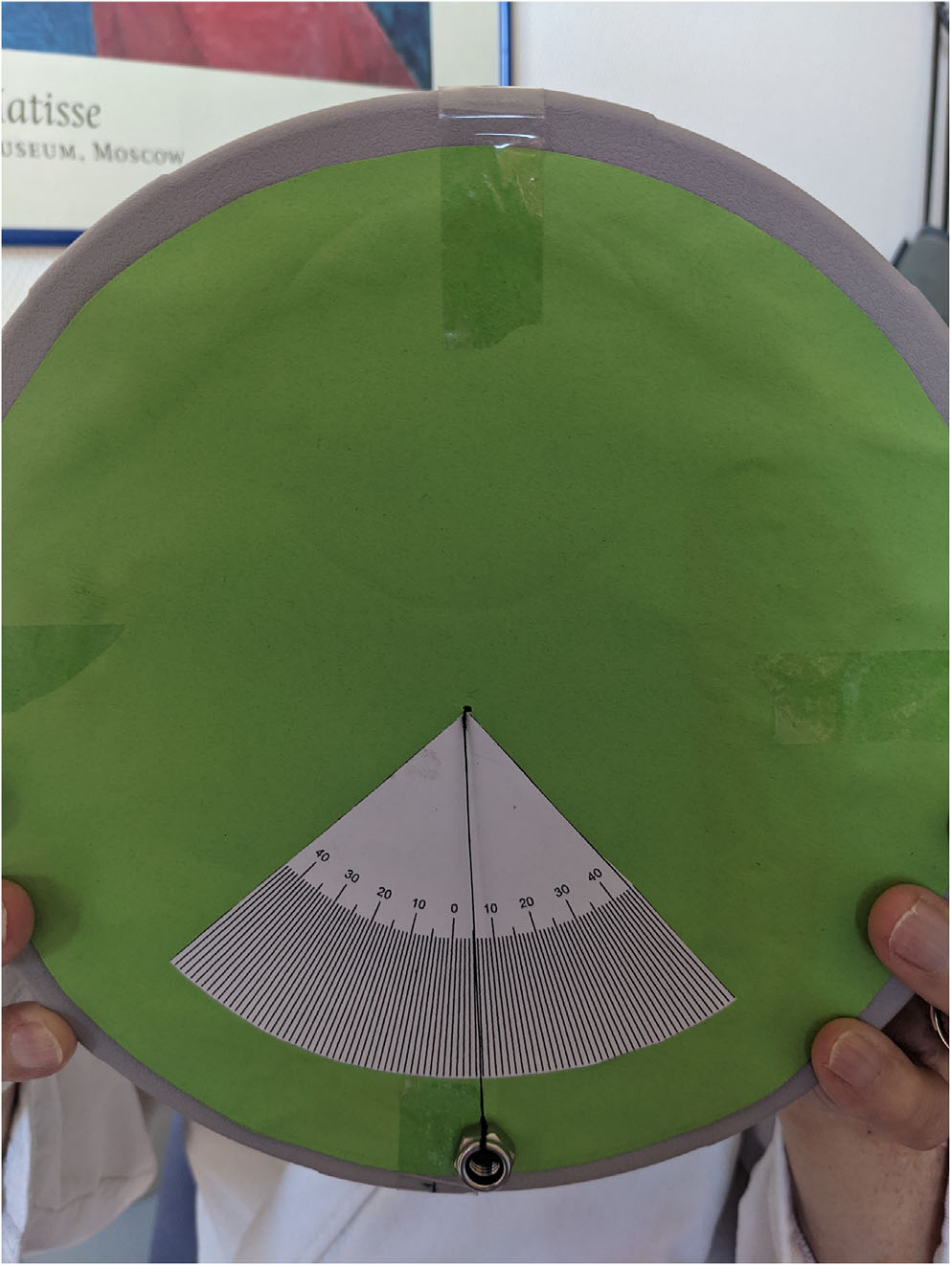
Example of a bucket test with an SVV tilt of five degrees to the left.

- Tilt of the subjective visual vertical axis is an important finding in patients presenting with acute gait and balance disorder. The main causes are acute unilateral peripheral vestibular deficits and cerebral (vascular) lesions, mostly in the brainstem. The side of the SVV tilt depends on the location of the lesion.
- What is interesting about this case is that although there is an ipsi‐ and contralateral lesion that could explain the SVV tilt, the clinical symptoms of lateropulsion and SVV tilt remain without an ocular tilt response or other symptoms.^1^ However, the lesion in the brainstem is the most likely reason in this case.
- The bucket test (see Fig. 2) is a valid, easy–to‐use, and useful test to examine the subjective vertical axis.^3^
- Training can improve the tilt of the SVV. Is it recommended to provide physiotherapy and the use of a walker as long as the SVV tilt is above 3°.

## Supporting information


Video S1.

**Caption S1**.
