# Supplementary material for: Case of a 96‐year‐old woman with tilt of the subjective vertical axis
Source: Ann Clin Transl Neurol. 2025 Feb 11;12(4):881–3. doi: 10.1002/acn3.70003 (PMC12040504; doi:10.1002/acn3.70003)
Supplement: Supplementary file 2 — Caption S1. [file ACN3-12-881-s001.docx]

The video shows the tilt of the subjective visual vertical axis at the admission to the neurogeriatric ward and at the discharge. At the time of admission, the patient is unable to walk independently. After rehabilitation on the ward, she is able to walk unassisted at discharge.
